# Salicylic Acid Mitigates Cadmium Stress in Wheat: Experimental Insights Into Growth and Biochemical Parameters

**DOI:** 10.1155/sci5/6887694

**Published:** 2024-11-30

**Authors:** Asma Zulfiqar, Beenish Gul, Ammara Saleem, Areeba Islam, Usman Zulfiqar, Muhammad Fraz Ali, Mohsin Nawaz, Abdullah Ahmed Al-Ghamdi, Humaira Rizwana

**Affiliations:** ^1^Institute of Botany, University of the Punjab Lahore, Lahore 54590, Pakistan; ^2^Department of Agronomy, Faculty of Agriculture and Environment, The Islamia University of Bahawalpur, Bahawalpur 63100, Pakistan; ^3^College of Agronomy, Northwest A&F University, Yangling, Xianyang 712100, China; ^4^Institute of Environment and Ecology, School of Environment and Safety Engineering, Jiangsu University, Zhenjiang 212013, China; ^5^Department of Botany and Microbiology, College of Science, King Saud University, P.O. 2455, Riyadh 11451, Saudi Arabia

**Keywords:** antioxidant defense, crop physiology, heavy metal stress, salicylic acid, wheat

## Abstract

The purpose of this study was to investigate the impact of salicylic acid (SA) on wheat subjected to cadmium (Cd) stress. The experiments were conducted during the winter season of 2022-2023 (November to February) at the University of the Punjab in Lahore, Pakistan. The study involved four wheat varieties: Akbar-2019, Galaxy-2013, Ujala-16, and Chakwal-86. The study utilized a factorial design with three replicates, examining three Cd levels (0.1 mM, 0.2 mM, and 0.3 mM) and two SA levels (0.5 mM and 0.9 mM). SA was applied as a seed priming agent, while cadmium sulfate (CdSO_4_) solution induced Cd toxicity. Various growth parameters, including plant height, total plant length, leaf length, leaf breadth, and leaf area, were measured alongside physiological and biochemical parameters such as total chlorophyll content, carotenoid content, oxidative stress indicators (MDA and H_2_O_2_), and antioxidants (total soluble protein, CAT, and APX)—to assess the effects of SA under Cd stress. The results indicated that the application of 0.5 mM SA resulted in the highest vegetative growth and maximum physiological and biochemical parameters, while 0.3 mM Cd significantly reduced growth. The performance of the treatments was observed in the following order: 0.5 mM SA > 0.3 mM Cd. Ujala-16 showed intermediate growth and yield, while Chakwal-86 had the lowest growth rate and yield. The study demonstrated that SA mitigates Cd stress effects, with 0.9 mM SA and 0.1 mM Cd yielding the highest growth, second only to 0.5- and 0.9-mM SA treatments. These findings underscore the potential of SA to enhance wheat growth and yield in Cd-contaminated soils. In conclusion, SA is suggested as a beneficial treatment for improving productivity and economic returns in Cd-stressed areas. Future recommendations include conducting long-term studies to evaluate cumulative treatment effects and investigating how salicylic acid mitigates cadmium stress through biochemical pathways and gene expression, enhancing agricultural practices.

## 1. Introduction

For the vast majority of people on the planet, wheat (*Triticum aestivum* L.) is a staple grain and the main source of nutrition [1]. In the human diet, cereals constitute the primary source of carbohydrates, the main source of energy, and a substantial supply of protein. Wheat grain has a higher protein content. Additionally, wheat is a great source of dietary fiber and a good supplier of both macro and micronutrients [2]. Plants thrive within a diverse array of environmental challenges, including both abiotic stresses, such as temperature fluctuations, heavy metal stress, drought stress, antibiotic pollution and soil conditions, and biotic stresses, such as pest infestations and disease pressures. A combination of significant, inorganic, radioactive, and natural metals can be found in the mechanical waste [3–5]. Soil provides a crucial environment for various forms of life, including plants, microorganisms, and animals [6]. It supports ecosystems and contributes to numerous ecological processes. Heavy metals are elements that can contaminate soil through various means, such as industrial activities, pollution, or improper waste disposal [7]. These metals have high densities (greater than 5 g per cubic centimeter), which is one way to classify them. The high density of these metals often correlates with their potential to be toxic in high concentrations [8]. Examples of heavy metals include lead, mercury, cadmium, and arsenic. When these metals accumulate in the soil, they can negatively impact plant growth, soil health, and the broader ecosystem [9]. Among all these heavy metals, Cadmium ions are highly soluble in water, which means they can easily dissolve and move through soil and water systems. This high solubility facilitates its spread and bioavailability, making it easier for cadmium to enter the food chain and affect living organisms [10]. Cadmium (Cd) has an extensive biological half-life. It develops in soil as a result of the weathering of the primary rocks and builds up as a result of human activity, including the accumulation of airborne Cd from smelting and mining, wastewater, soil amendments, drainage, and the use of phosphate fertilizers contaminated with Cd [11]. Soils are becoming increasingly contaminated with salts of toxic heavy metals, which causes dehydration in plants as well as a decline in yield and quality [12]. High concentrations of metals like cadmium can't be degraded and have detrimental effects on plants. Cadmium stress directly harms plant cells by inhibiting cytoplasmic enzymes and causing oxidative damage to cell components [13]. Higher cadmium (Cd) uptake adversely affects photosynthesis in plants, leading to metal accumulation in foliage, impaired chloroplast membranes, changes in leaf tissue, reduced photosynthetic pigments, altered cytoplasmic enzymes, and damage to enzymes involved in carbon reduction and the xanthophyll cycle [14].

Plants employ many defense strategies to fend off the damaging effects of Cd, such as attaching Cd to the cell wall, chelating with phytochelatins (PCs) to retain vacuoles, and boosting the antioxidant system [15]. Priming seeds with phytohormones is a popular technique to enhance seed quality. This method improves germination rates, leading to higher resistance to biotic and abiotic stresses and increased agricultural yields. Phytohormones, particularly salicylic acid (SA), offer a cost-effective solution for promoting safe crop production in cadmium-contaminated soil and have been proven effective across various crops [16]. Salicylic acid (SA) navigates biochemical pathways to regulate growth and development by orchestrating signal transductions, especially in response to stressful conditions. A straightforward phenolic molecule called SA has been known to be a powerful phytohormone that controls growth and protection in plants [17]. Two separate and compartmentalized pathways are used to synthesize SA. One process originates from phenylalanine and occurs in the cytosol. Another biosynthetic route is the one in which chorismate is transformed into isochorismate through an enzyme called isochorismate synthase [18]. In recent years, numerous plant species have shown the beneficial benefits of SA on increasing their capacity to withstand Cd [19]. Catalase (CAT) and ascorbate peroxidase (APX) are impacted by SA's mechanism of action [20]. Previous study showed the specific mechanisms underlying the reduction of cadmium (Cd) accumulation in rice grains through the application of salicylic acid (SA) spray. Their research revealed that SA treatment effectively mitigated Cd toxicity in rice plants without altering factors such as pH levels, total or accessible Cd content, or the activities of antioxidant enzymes responsible for reducing hydrogen peroxide (H_2_O_2_) accumulation [21]. Consequently, the application of SA spray on wheat emerges as a promising strategy for addressing Cd contamination in wheat fields [22]. Previous studies have shown that wheat can mitigate heavy metal stress by boosting oxidative stress responses and facilitating the compartmentalization of metals in vacuoles. Nonetheless, effective methods to enhance cadmium (Cd) tolerance and limit Cd accumulation in various wheat cultivars remain uncertain. This research aims to assess how salicylic acid (SA) can improve Cd tolerance, determine the most effective concentration of SA for application, and investigate the antioxidant mechanisms through which SA affects Cd tolerance and detoxification in wheat. The results will deepen our understanding of SA's role in enhancing Cd tolerance and support the safe cultivation of wheat by increasing growth and antioxidant potential in soils contaminated with Cd.

## 2. Materials and Methods

### 2.1. Experimental Design

The experiments were conducted during the winter season of 2022-2023 (November to February) at the University of the Punjab in Lahore, Pakistan. The study involved four wheat varieties: Akbar-2019, Galaxy-2013, Ujala-16, and Chakwal-86. Certified seeds of wheat were collected from Punjab Seed Corporation, Lytton Road, Lahore. Before sowing, wheat seeds were sterilized with bleaching agents (1% HgCl_2_). The experiment was conducted in Botanical Garden of Quaid-e-Azam Campus, University of the Punjab in Lahore. The soil of the Botanical Garden was tested at the Soil and Water Testing Laboratory in Lahore. The impact of salicylic acid (SA) and cadmium (Cd) on wheat was examined in the experiment. Three levels of Cd (0.1 mM, 0.2 mM, and 0.3 mM) and two levels of SA (0.5 mM and 0.9 mM) were applied in the form of solution, with SA administered as a seed priming agent. The experiment followed a factorial design with the following treatment combinations: control C0 (no Cd, no SA), Cd 1(Cd 0.1 mM), Cd 2 (0.2 mM), Cd3 (Cd 0.3 Mm), SA1 (SA 0.5 Mm), SA2 (SA 0.9 Mm), SA1Cd1(Cd 0.1 mM + SA 0.5 mM), SA2Cd1(Cd 0.1 mM + SA 0.9 mM), SA1Cd2 (Cd 0.2 mM + SA 0.5 mM), SA2Cd2(Cd 0.2 mM + SA 0.9 mM), SA1Cd3 (Cd 0.3 mM + SA 0.5 mM), and SA2Cd3 (Cd 0.3 mM + SA 0.9 mM). Each treatment was replicated three times to ensure the reliability of the results. SA was administered as a seed priming agent by soaking the seeds in the SA solutions for 24 h before sowing. The experiment duration was 8 weeks. The plants were grown in a wire house under natural environmental conditions, with temperatures ranging from 25°C to 30°C and a consistent watering regime. Various morphological parameters were measured to assess the effects of the treatments.

### 2.2. Data Collection and Harvesting

After 8 weeks, four seedlings were carefully uprooted and rinsed with distilled water to remove any surface dust and residues. One month later, leaf and root samples from each treatment group were collected for the analysis of photosynthetic, oxidative stress and antioxidants. The leaves were cleaned with distilled water, immediately frozen in liquid nitrogen, and stored at −80°C for subsequent analysis.

### 2.3. Morphological Parameters

Plant height, total plant length, leaf length and leaf width were measured with scale. Carleton and Foote [23], protocol was used for the measurement of leaf area (cm^2^) using formula.

(1) $$\begin{array}{c} \text{Area of leaf}=\text{leaf length}\times \text{leaf width}\times 0.75. \end{array}$$

### 2.4. Fresh and Dry Weight of Root and Shoot

Using a weighing balance, the fresh weight of the root and shoot was determined. Then placed in petri plates and dried in a drying oven. The drying oven was set at 70°C. The oven dried samples were taken and their dry weights were recorded. Relative water content was determined by the procedure of González and González-Vilar [24]. Weight (Wf) of fresh leaves was measured after they were removed from the plants. In petri dishes, the leaves were then submerged in distilled water for a whole day. The leaves were weighed after being removed from the water and patted dry with blotting paper for a day. The leaf's turgid value (Wt) was measured. Following that, the leaves were oven dried for 24 h at 70°C. One more measurement was made of the weight of the leaves which is their dry weight (Wd).

(2) $$\begin{array}{c} \text{Relative water content }\%=\left[\frac{\left(\text{Wf}–\text{Wd}\right)}{\left(\text{Wt}–\text{Wd}\right)}*\left(100\right)\right]. \end{array}$$

### 2.5. Photosynthetic Parameters

After dipping 0.5 g of leaves in 5 mL of DMSO in falcon tubes and letting them in the dark for 48 h, the absorbance of the extracts was measured using a Shimadzu UV-1800 Spectrophotometer at 645 nm and 663 nm. A formula given by Arnon [25] was used to calculate the total chlorophyll content;

(3) $$\begin{array}{c} \text{Total Chlorophyll Content }\left(\mu g\;g^{-1}\text{FW}\right)=\left(0.00802\right)\left(D.F.-663\right)+\left(0.0202\right)\left(D.F.-645\right)\left(\text{mL of solvent}\right). \end{array}$$

The extracts' absorbance was measured for carotenoids concentration at 480 nm using a Shimadzu UV-1800 spectrophotometer. The amount of carotenoids was calculated using the formula provided by Wellburn [26], which is:

(4) $$\begin{array}{c} \text{Carotenoids Content}=\left\{\left(1000\right)\left(480-1.29\right)\left(D.F.-663\right)–53.78\;\frac{\left(D.F.-645\right)}{220}\right\}. \end{array}$$

### 2.6. Determination of Antioxidant Activity

#### 2.6.1. Extraction of Enzyme

The mortar was filled with 0.2 g of fresh leaves. Next, the tissue was fully submerged in liquid nitrogen, using an appropriate amount. With the aid of a pestle and mortar, leaves were homogenized in liquid nitrogen until the nitrogen nearly evaporated and the tissue ground into a powder. Grinded for several minutes in a cooled mortar, 1.5 mL of 150 mM phosphate buffer with pH 7 was added to the crushed tissue. Concentrate was spun at 12,000 rpm (4°C) for 20 min. Supernatant should be decanted into Eppendorf tubes.

### 2.7. Total Soluble Protein

In test tubes, 3 mL of color reagent (Commassie Brilliant Blue G-250) was added to 100 μL of extraction solution to determine the total soluble protein. After giving the reaction mixture a good vortex for a minute, it was given 5 minutes to incubate. The solution's absorbance at 595 nm was measured using the Bradford method, employing a spectrophotometer (Shimadzu, UV-1800) [27].

### 2.8. Catalase Activity

With only minor modifications, CAT activity has been measured using the methodology provided by Chance and Maehly [28]. The reaction was started with a 3 mL reaction mixture that contained 1.9 mL PBS, 1 mL H_2_O_2_, and 100 μL enzyme extract. After 10 s, changes in absorbance were seen at 240 nm for 60–80 s.

### 2.9. Ascorbate Peroxidase

With few modifications, the Nakano and Asada [29] approach has been used to measure APX activity. 2.75 mL of sodium acetate buffer, 50 μL EDTA, 50 μL H_2_O_2_, 50 μL ascorbic acid, and 100 μL of extracted enzyme solution made up the reaction mixture. Using a UV spectrophotometer (Shimdazu UV-1800), the decrease in absorbance was measured at 390 nm every 10 s for 60–80 s.

### 2.10. Determination of Oxidative Stress

The method of Jana and Choudhuri [30] was followed in order to calculate the amount of H_2_O_2_. Fresh leaf samples were extracted using a solution containing 0.1% titanium sulphate and 1 mL of H_2_SO_4_. Spectrophotometric measurements of the absorbance were made at 410 nm wavelength following a 20-min centrifugation at 6000 rpm.

To measure lipid peroxidation, the amount of malondialdehyde (MDA) was determined. Samples of seedlings were extracted using a buffer solution (50 mM, pH 7.8) that included 1% polyethylene pyrrole. After that, the mixture was centrifuged for 20 min at 10,000 rpm and 4°C. As suggested by Heath and Packer [31], the absorbance was determined using the extinction coefficient value of 155 nM^−1^·cm^−1^, and the MDA content was determined using the corrected optical density.

### 2.11. Yield Parameters

The number of grains per plant were counted and their fresh and dry weight was weighed using a weighing balance.

### 2.12. Statistical Analysis

The SPSS software application, version 20, was used to do the statistical analysis of the data. To test for significant differences (*p* < 0.05), the data was analyzed using ANOVA two way and POST HOC commands for varieties and treatments. Duncan's Multiple Range Test was used to rank the treatments. Treatments, varieties, and their interactions were considered fixed factors in the data analysis, while replications were considered random factors. As a result were grouped into tables and graphs that illustrated both the individual behavior and the link between them. Moreover, Pearson correlation analysis was employed to assess the relationships between the different variables under scrutiny, with computations conducted using RStudio.

## 3. Results

### 3.1. Impact of SA (Salicylic Acid) on Morphological Parameters Under Cd Stress

The application of SA had a beneficial effect on plant growth. The findings clearly show that SA treatment, compared to the control group, resulted in significant improvements in plant height, total plant length, leaf length, leaf width, and leaf area (Supporting Table 1). Among the treatments, 0.5 mM SA exhibited the most substantial increase in growth parameters, highlighting its positive impact on wheat growth and productivity. In contrast, Cd exposure had a detrimental effect on plant growth, but SA effectively mitigated Cd-induced stress. Specifically, Akbar-2019 showed the highest growth among all varieties. At 0.5 mM SA, Akbar-2019 exhibited a 40.7% increase in plant height compared to the control group. Leaf length, leaf width, and leaf area also increased by 52%, 26%, and 90%, respectively, relative to the control. Additionally, Akbar-2019 outperformed Chakwal-86, with a 51% increase in plant height, 50% in leaf length, 19% in leaf width, and 78% in leaf area. However, 0.9 mM SA in combination with 0.1 mM Cd was more effective in mitigating Cd stress, with Akbar-2019 showing a 31% increase in plant height compared to the control. Similar trends were observed for other growth parameters. Although plant growth decreased at 0.9 mM SA and 0.3 mM Cd due to higher Cd concentration, it remained higher than untreated plants. As Cd concentration increased, plant growth was increasingly inhibited due to heavy metal stress.

### 3.2. Impact of SA (Salicylic Acid) on Plants Biomass Under Cd Stress

The application of salicylic acid (SA) had significant effects on both fresh and dry weights of shoots and roots (Supporting Table 2). At 0.5 mM SA, the Akbar-2019 variety showed a notable increase in fresh shoot weight, approximately 3.8% higher than Chakwal-86. The fresh shoot weight of Ujala-16 was about 3.7% higher than Chakwal-86, with Akbar-2019 still exhibiting the highest fresh shoot weight. However, at a higher concentration of 0.9 mM SA, all varieties experienced a reduction in shoot weight, though Akbar-2019 maintained a relatively higher fresh shoot weight with a 3.08% increase compared to Chakwal-86.

**Figure 1:**
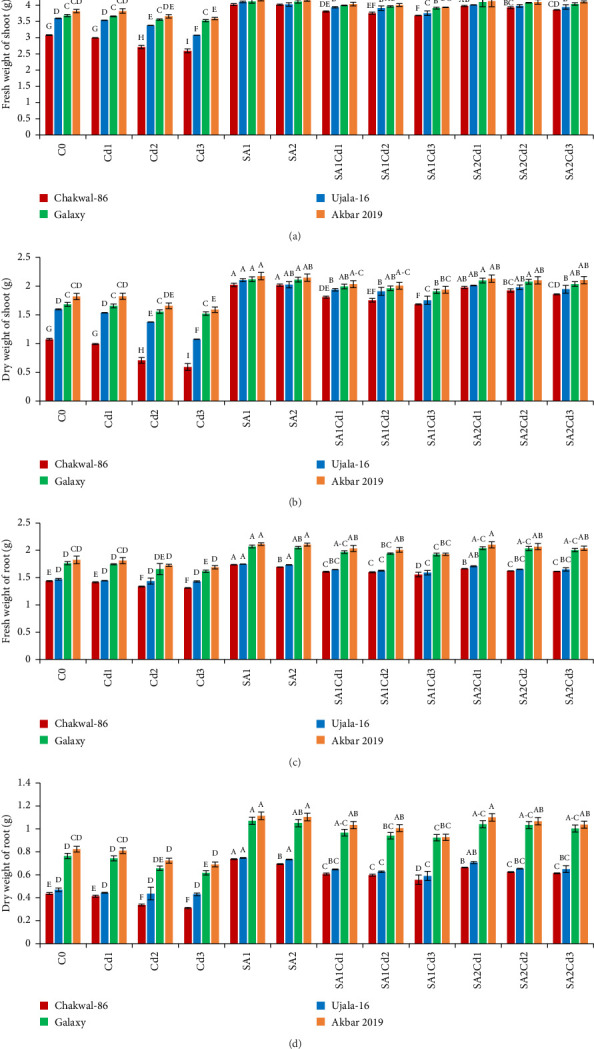
Effects of different doses of SA and Cd on biomass of *T. aestivum* (cultivars): fresh weight of shoot (a), dry weight of shoot (b), fresh weight of root (c), and dry weight of root (d). With the use of Duncan's multiple comparison test, statistical significance is indicated by distinct upper-case letters above the bars. In the figures, the data is the mean of three repeats (*n* = 3), with error bars representing the standard deviation (SD) of three repeats.

At 0.5 mM SA, Akbar-2019 also demonstrated substantial increases in both fresh and dry weights of shoots and roots, with increases of 9% and 19% for shoots and 16% and 35% for roots, respectively, compared to the control group. Notably, a higher concentration of 0.9 mM SA proved more effective in alleviating stress and promoting greater weights. Under Cd stress (0.1 mM Cd with 0.9 mM SA), Akbar-2019 achieved increases in fresh and dry weights of shoots and roots by approximately 4% and 8% for shoots, and 26% and 67% for roots, respectively, surpassing Chakwal-86 (Figure 1).

### 3.3. Impact of SA (Salicylic Acid) on Relative Water Content Under Cd Stress

**Figure 2:**
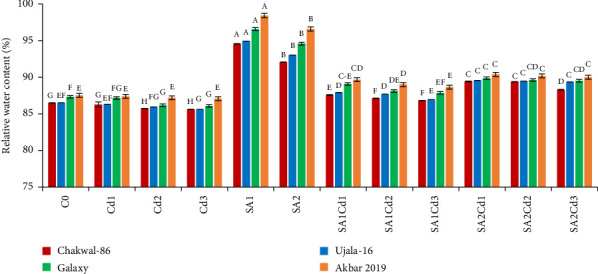
Effects of different SA and Cd treatments on the relative water content of *T. aestivum* (cultivars). Duncan's multiple comparison test was used to evaluate statistical significance, which is shown by distinct upper-case letters above the bars. The data shown in the figures is the mean of three repeats (*n* = 3), whereas error bars show the standard deviation (SD) of three repeats.

The study of Relative Water Content (RWC) across different cultivars and treatments highlights distinct plant responses to stress (Supporting Table 2). Chakwal-86 maintains a relatively stable RWC across treatments, with the highest RWC of 94.55% observed under SA1 treatment, demonstrating a strong response to salicylic acid. Although cadmium stress significantly reduces its RWC, salicylic acid treatments help in partial recovery. Ujala-16 shows a similar trend, with its maximum RWC reaching 94.91% under SA1, indicating substantial benefits from salicylic acid. While cadmium stress reduces its RWC, the decrease is less pronounced than in Chakwal-86. Galaxy-2013 displays the highest RWC overall, especially under SA1 treatment with 96.58%, showcasing exceptional resilience to both cadmium and salicylic acid. Akbar-2019 records the highest RWC among all cultivars, peaking at 98.41% under SA1, highlighting its superior tolerance to stress and efficiency in maintaining water content (Figure 2).

### 3.4. Impact of SA (Salicylic Acid) on Photosynthetic Parameters Under Cd Stress

**Figure 3:**
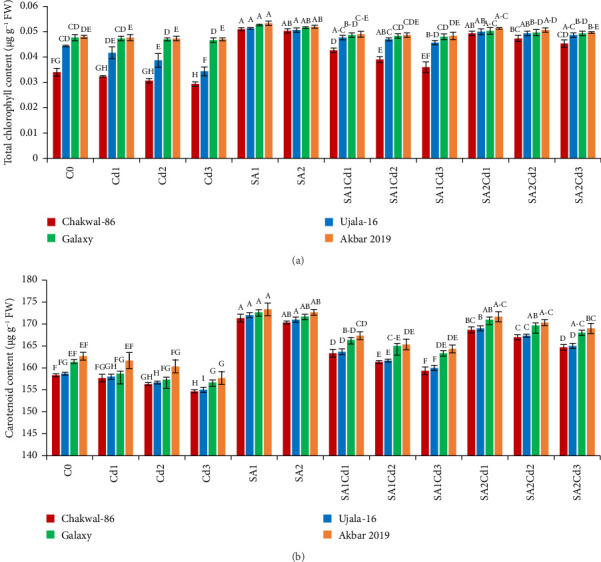
Impact of various SA and Cd treatments on photosynthetic parameters of four cultivars of *T. aestivum*. (a) Total chlorophyll content. (b) Carotenoid content. Distinct upper-case letters above the bars represent statistical significance, which was determined using Duncan's multiple comparison test. Error bars indicate the standard deviation (SD) of three repeats, while the data displayed in the figures is the mean of three repeats (*n* = 3).

The application of salicylic acid (SA) significantly increased the total chlorophyll and carotenoid content in *Triticum aestivum* (Supporting Table 2). Conversely, cadmium (Cd) had a negative impact on photosynthetic parameters. Among the wheat varieties studied, Akbar-2019 exhibited notably higher chlorophyll and carotenoid content compared to Galaxy-2013, Ujala-16, and Chakwal-86. Specifically, when treated with 0.5 mM of SA, Akbar-2019 showed the highest chlorophyll content, with increases of 1.3%, 4%, and 5% compared to Galaxy-2013, Ujala-16, and Chakwal-86, respectively. The carotenoid content of Akbar-2019 treated with 0.5 mM of SA was also 6% higher compared to the control. Furthermore, at 0.9 mM of SA combined with 0.1 mM Cd stress, Akbar-2019 exhibited a 5% increase in carotenoid content compared to untreated plants. Similarly, at 0.5 mM of SA along with 0.1 mM Cd, it showed nearly a 3% enhancement in carotenoid content compared to the control. These findings suggest that 0.9 mM of SA under Cd stress is more effective in alleviating stress than 0.5 mM of SA under Cd stress, as depicted in Figure 3. This study highlights the beneficial role of SA in enhancing photosynthetic pigments and mitigating the adverse effects of Cd stress in wheat, underscoring its potential application in agricultural practices to improve crop resilience and productivity.

### 3.5. Impact of SA (Salicylic Acid) on Antioxidant Activity Under Cd Stress

**Figure 4:**
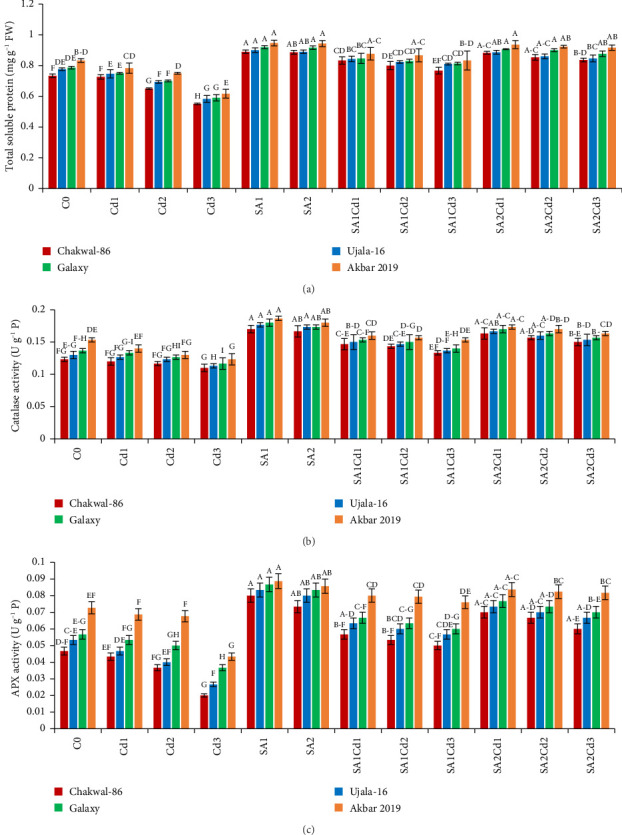
The influence of numerous treatments of SA and Cd on total soluble protein antioxidant enzyme activities and oxidative stress in *T.aestivum* cultivars, namely total soluble protein (a), catalase activity (b) and APX activity (c). Distinct upper-case letters above the bars represent statistical significance, which was determined using Duncan's multiple comparison test. Error bars indicate the standard deviation (SD) of three repeats, while the data displayed in the figures is the mean of three repeats (*n* = 3).

The antioxidant potential of the cultivars under different treatments exhibits distinct patterns (Supporting Table 3). Chakwal-86 shows a general increase in antioxidant enzyme activities with salicylic acid (SA) treatments compared to cadmium (Cd) stress alone. Specifically, catalase (CAT) and ascorbate peroxidase (APX) activities peak under 0.5 mM SA treatment (SA1), with values reaching 0.17 and 0.08 U·g^−1^ P, respectively. Ujala-16 also shows improved antioxidant activities under SA treatments, with CAT reaching 0.18 U·g^−1^ P and APX at 0.083 U·g^−1^ P, while MDA and H_2_O_2_ levels decrease significantly, showing enhanced stress tolerance. Galaxy-2013 presents a similar trend, with maximum CAT and APX activities of 0.18 and 0.0867 U·g^−1^ P under SA1, respectively, and reduced oxidative markers, suggesting strong antioxidant defense. Akbar-2019 exhibits the highest antioxidant activities overall, particularly under SA1, where CAT and APX reach 0.187 and 0.089 U·g^−1^ P, respectively. This cultivar effectively minimizes oxidative damage, reflected in its lowest MDA and H_2_O_2_ levels under SA treatments, highlighting its superior capacity to counteract stress-induced oxidative stress (Figure 4).

### 3.6. Impact of SA (Salicylic Acid) on Oxidative Stress Under Cd Stress

**Figure 5:**
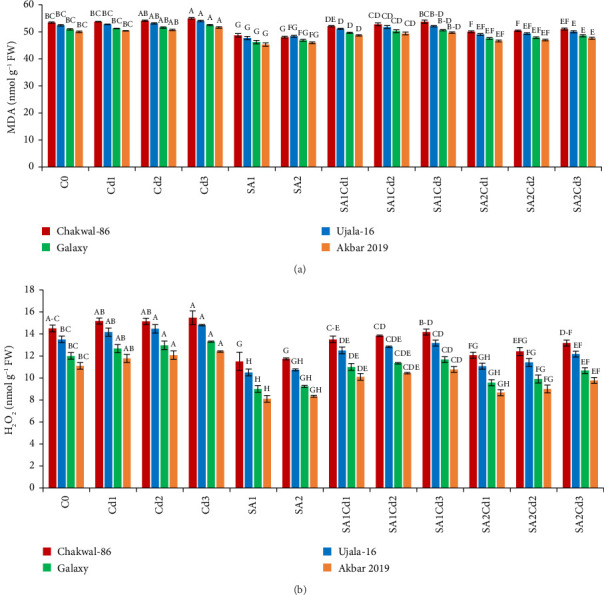
The impact of multiple SA and Cd treatments on oxidative stress in cultivars of *T.aestivum*, MDA (a) and H_2_O_2_ (b). Duncan's multiple comparison test was used to evaluate statistical significance, which is shown by distinct upper-case letters above the bars. The data shown in the figures is the mean of three repeats (*n* = 3), whereas error bars show the standard deviation (SD) of three repeats.

Treatment with 0.5 mM salicylic acid (SA) resulted in a significant reduction in lipid peroxidation in the wheat variety Akbar-2019, as evidenced by approximately 9% decrease in malondialdehyde (MDA) levels in its leaves compared to the control. Conversely, Chakwal-86 exhibited a notable increase in MDA levels, with a rise of approximately 7%, indicating heightened oxidative stress. Among all varieties, the highest level of oxidative stress was induced by 0.3 mM cadmium (Cd), with the stress severity ranked as follows: Chakwal-86 > Ujala-16 > Galaxy-16 > Akbar-2019, with Akbar-2019 demonstrating the lowest oxidative stress. Similarly, hydrogen peroxide (H_2_O_2_) levels mirrored this trend. Akbar-2019 displayed a 22% reduction in H_2_O_2_ levels when treated with 0.9 mM SA and 0.1 mM Cd relative to the control. In contrast, Chakwal-86 exhibited a 39% increase in oxidative stress compared to Akbar-2019, highlighting its greater susceptibility (Supporting Table 3; Figure 5).

### 3.7. Impact of SA (Salicylic Acid) on Yield Parameters Under Cd Stress

**Figure 6:**
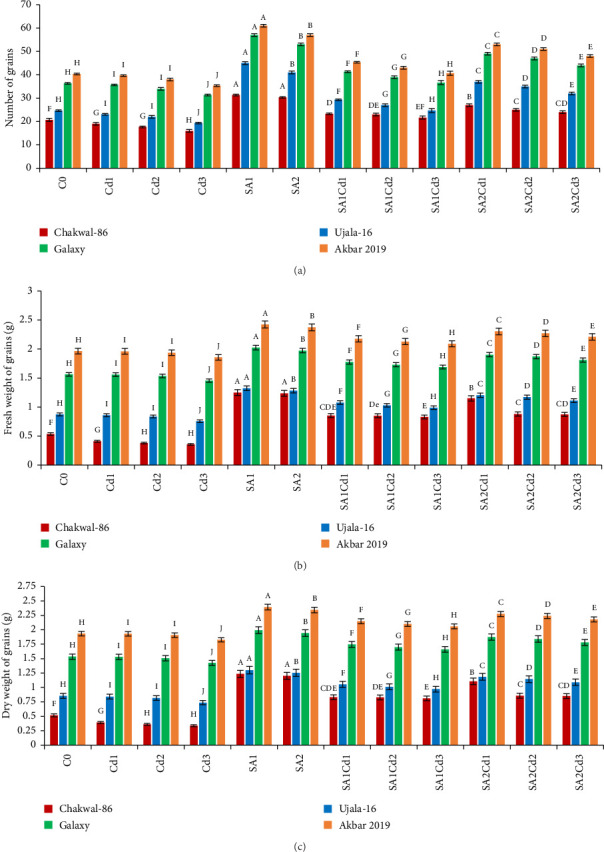
Effects of different SA and Cd treatments on four *T. aestivum* cultivars' yields. (a) The number of grains. (b) Grain fresh weight. (c) Grain dry weight. Duncan's multiple comparison test was used to evaluate statistical significance, which is shown by distinct upper-case letters above the bars. The data shown in the figures is the mean of three repeats (*n* = 3), whereas error bars show the standard deviation (SD) of three repeats.

Analysis of grain production across various treatments for different wheat cultivars reveals distinct trends (Supporting Table 3). Chakwal-86 exhibited a significant increase in both grain number and weight with salicylic acid (SA) treatments, achieving the highest values of 31.33 grains and 1.25 g under 0.5 mM SA. In contrast, cadmium (Cd) stress led to a substantial reduction in these parameters, with the lowest grain count of 16 grains and weight of 0.357 g under 0.3 mM Cd. Similarly, Ujala-16 demonstrated enhanced grain production under SA treatments, peaking at 45 grains and 1.323 g with 0.5 mM SA. However, Cd stress reduced grain production, with the lowest values of 19.33 grains and 0.757 g observed under 0.3 mM Cd. Galaxy-2013 achieved the highest overall grain production, recording 57 grains and 2.023 g under 0.5 mM SA, although it also experienced reductions under Cd stress, with the lowest values being 31.33 grains and 1.457 g under 0.3 mM Cd. Akbar-2019 exhibited the highest grain counts and weights, reaching 61 grains and 2.423 g under 0.5 mM SA. Nonetheless, it showed a significant decline in grain production under Cd stress, with the lowest values of 35.33 grains and 1.857 g under 0.3 mM Cd. Overall, SA treatments consistently enhanced grain production across cultivars, whereas Cd stress negatively impacted all cultivars, with Akbar-2019 demonstrating the greatest resilience and productivity (Figure 6).

### 3.8. Pearson Correlation for Various Parameters Across Four Cultivars of *T. aestivum*

**Figure 7:**
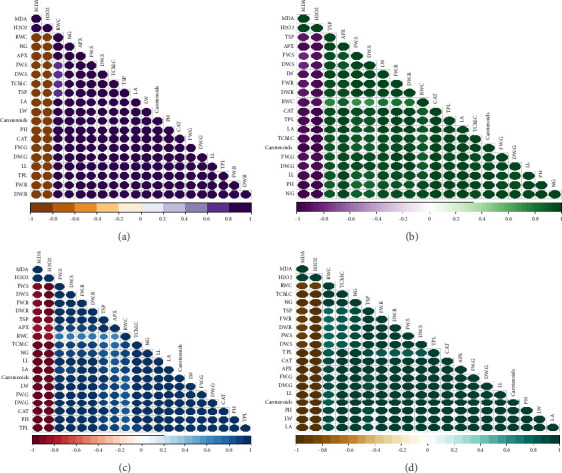
Pearson correlation for four *T. aestivum* cultivars' (a) (Ujala-16), (b) (Chakwal-86), (c) (Akbar-2019), and (d) (Galaxy-2013). Different abbreviations used in this figure are as follow: APX, ascorbate; CAT, catalase; DE. S, shoot dry weight; DW. G, grains dry weight; DW. R, root dry weight; FW. G, grains fresh weigh; FW. R, root fresh weight; FW. S, shoot fresh weight; H_2_O_2_, Hydrogen peroxide; LA, leaf area; LL, leaf length; LW, leaf area; MDA, *Malondialdehyde*; NG, number of grains; PH, plant height; RWC, relative water content; TChl C, total chlorophyll content; TPL, total plant length; TSP, total soluble protein.

In our study, the Pearson correlation coefficient served as a crucial statistical tool for assessing the strength and direction of linear relationships between variables. Specifically, it allowed us to investigate the interplay among various parameters across four distinct cultivars of wheat. As per our findings, a correlation coefficient of −1 denotes a perfect negative linear relationship, indicating that as one variable increases, the other variable decreases proportionally. Conversely, a correlation coefficient of 0 signifies the absence of a linear relationship between the variables (Figure 7).

## 4. Discussion

In the proposed study, the wheat variety Akbar-2019 exhibited significantly higher values for both morphological and biochemical parameters compared to other varieties. Interestingly, optimal growth and production were observed across all varieties with salicylic acid (SA) at 0.5 mM, contrasting with reduced effectiveness under cadmium (Cd) stress conditions. SA demonstrated greater efficacy than under Cd stress, as illustrated in the results. Treatments with SA alone and SA under Cd stress yielded superior results in seedling growth, vegetative growth, and biochemical attributes. Notably, increasing SA concentration alone adversely affected all parameters, whereas the same increase under Cd stress positively influenced all evaluated parameters.

**Figure 8:**
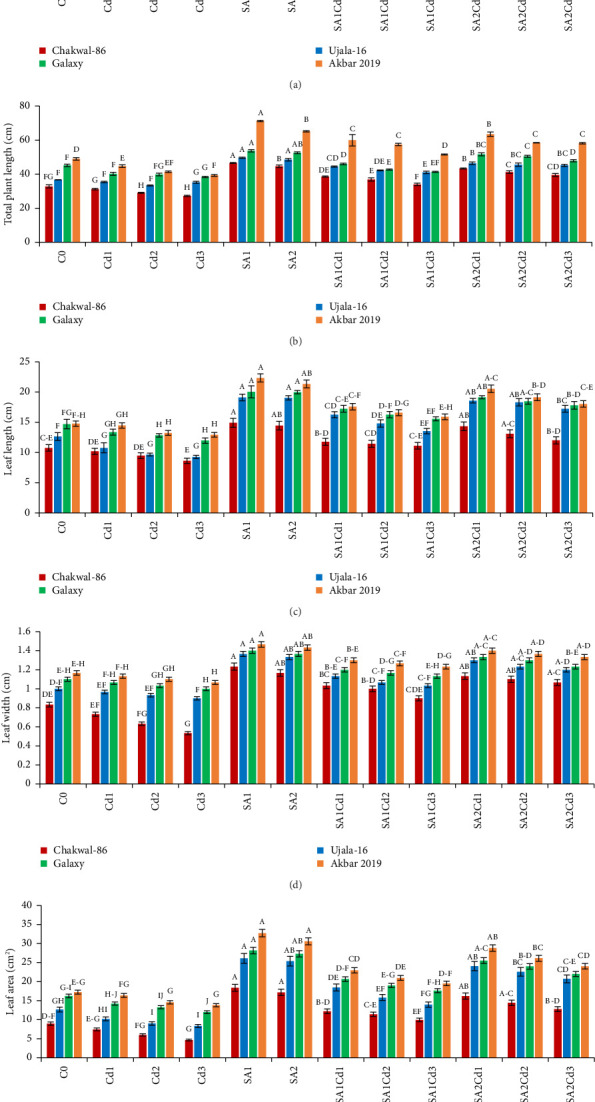
Various SA and Cd treatments' effects on *T. aestivum* (cultivars) morphological parameters: plant height (a), total plant length (b), leaf length (c), leaf width (d) and leaf area (e). Distinct upper-case letters above the bars represent statistical significance, which was determined using Duncan's multiple comparison test. Error bars indicate the standard deviation (SD) of three repeats, while the data displayed in the figures is the mean of three repeats (*n* = 3).

The use of SA was found to increase the morphological characteristics, but Cd had negative impacts on plant growth. Most importantly wheat variety Akbar-2019 exhibited the highest growth than all other varieties. By limiting leaf photosynthesis, lowering gas exchange properties, and changing how wheat absorbs minerals and nutrients, Cd toxicity may reduce wheat growth and biomass. The decrease in wheat growth and biomass caused by Cd could be attributed to many physiological and molecular toxicity pathways in wheat [32]. The reduction in IAA and cytokinin concentration may be the cause of the reduction in morphological characteristics brought on by Cd. According to previous study abiotic stress such as antibiotics [33], drought, salinity and heave metals i.e., cadmium, chromium, arsenic, nickel or lead adversely affect the morphological characteristics, photosynthetic pigments [34], and nutritional intake [35]. Pretreatment of seeds by SA boosted plant height and fresh/dry weights under both stress and non-stress conditions, which is consistent with research by [36]. By protecting the enzymes impacted by Cd stress, SA application enhances morphological characteristics (Figure 8). The application of Cd had a considerable impact on the leaf area, whereas plants treated with SA had more leaf area than control, these findings are align with [17, 22]. Leaf area can have a significant role in determining a plant's productivity because it is directly correlated with light absorption. Under Cd stress, the SA growth-inducing capabilities have been seen in rice and wheat [37] as well as barley [38]. Growth suppression is a common reaction of plants to cadmium stress [39]. Numerous studies have demonstrated the efficiency of using SA to reduce cadmium toxicity in various plant species. Plant growth is known to be regulated by a hormonal system that responds to environmental changes [40, 41].

Under Cd stress, plant biomass is decreased, but SA increases plant biomass. SA can modulate the synthesis and signaling of plant growth hormones such as auxins, cytokinins, and gibberellins. This regulation promotes cell division, elongation, and differentiation, which are essential processes for biomass accumulation [42]. When Cd stress was amplified, the weight of the stem fell, and using SA caused it to increase. As a result, with increased SA, a greater increase in fresh and dry weight of the stem under Cd stress was seen. The increase in ion leakage observed with salicylic acid (SA) application under salt stress can paradoxically contribute to increased plant productivity [43]. The fall in cell division and elongation causes a reduction in the biomass of the entire plant. This reduction may be brought on by the Cd effect, which may be due to inhibition of the proton pump [44]. Additionally, the negative effects of cadmium on wheat plants may also be caused by altered protein structures, subcellular distribution of Cd, and excessive synthesis of signaling molecules. Plant biomass, root length, and shoot length have all been utilized as markers of heavy metal toxicity. Previous study has found that plant growth, dry weight, shoot height and photosynthesis were found to be very sensitive to Cd [45].

Cd toxicity delays the normal physiological and biochemical of plants [46]. The overall chlorophyll content and carotenoid content in this study exhibit the similar pattern. Plants under Cd stress exhibit a reduction in both total chlorophyll content and carotenoid concentration, whereas SA relieves the stress and exhibits maximal photosynthetic parameters both on its own and in Cd-stressed soil. The enlarged leaf area observed in plants treated with SA may be attributed to a decrease in chlorophyll concentration and subsequent suppression of photosynthesis under Cd stress, which typically inhibits plant growth. The inhibition of photosynthetic activity in the leaf could be due to damage to the PSII response centre induced by cadmium application in rice [47]. Damage to PSII can impair its ability to efficiently transfer electrons and generate ATP and NADPH, which are essential for the light-dependent reactions of photosynthesis [48]. The decrease in photosynthetic pigments may be caused by Cd-mediated damage to the leaf photosystem's chloroplast structure and function. Chlorophyll is degraded and its biosynthesis is inhibited by cadmium stress, which can also lower photosynthetic efficiency and the rate of CO_2_ assimilation [49]. The lack of numerous critical minerals that are involved in the formation of chlorophyll pigments may be the cause of the drop in chlorophyll a and b [50]. A decrease in photosynthetic pigments in wheat seedlings was reported by [51], while another study reported a similar decline in cucumber (*Cucumis sativus*) plants exposed to Cd. Heavy metal stress such as Cd stress on plant chlorophyll pigments has a deleterious impact on photosynthesis. Plants' carotenoid concentrations decreased as a result of the elevated Cd content [52]. The influence on growth and photosynthetic rate may be the main cause of the growth suppression caused by Cd. Salicylic acid (SA) is known to induce various physiological responses in plants, including an increase in pigment concentration such as chlorophyll. This increase in chlorophyll content can have significant implications for photosynthetic enzyme activity [53]. Salicylic acid is a plant hormone that can enhance chlorophyll biosynthesis or stabilize chlorophyll molecules, leading to an increase in chlorophyll concentration in leaves [54]. Pretreatment of plants with salicylic acid (SA) prior to cadmium (Cd) exposure may mitigate Cd toxicity on carboxylating enzymes, potentially leading to an increase in total chlorophyll content in the presence of SA [55].

Our study demonstrated that plants treated with salicylic acid (SA) exhibited higher relative water content compared to plants stressed by cadmium (Cd), which showed lower relative water content. This difference can be attributed to the oxidative stress induced by reactive oxygen species (ROS), which damage cell membranes and disrupt cellular functions in Cd-stressed plants, leading to reduced water retention [56]. Cadmium (Cd) can hinder water absorption and transportation in plants by compromising the integrity and functionality of their root systems. This impairment reduces the plant's ability to absorb water from the soil. Cd poisoning leads to a decrease in relative water content, indicating an imbalance in water regulation. Interestingly, priming SA seeds with Cd beforehand has been observed to protect their relative water content [19, 57].

SA treatment has been observed to enhance plants' defense mechanisms. Conversely, cadmium (Cd) retards development and exacerbates oxidative damage. The synthesis of reactive oxygen species (ROS) is heightened with increasing Cd concentrations, prompting an increase in the production of antioxidant enzymes by the plant to counteract these free radicals. Despite this response, the antioxidant activity, including total soluble protein, catalase (CAT), and ascorbate peroxidase (APX), is diminished under Cd stress. Cadmium (Cd) impacts wheat's antioxidant enzymes such as total soluble protein, catalase (CAT), and ascorbate peroxidase (APX). Simultaneously, Cd induces the production of protective compounds while triggering oxidative stress within the plant [11]. Cadmium (Cd) has been observed to elevate malondialdehyde (MDA) levels in plants, primarily through the increased generation of hydrogen peroxide (H_2_O_2_) induced by Cd exposure. This phenomenon is consistently observed in both flax and chickpea plants treated with Cd, leading to heightened MDA levels and greater membrane permeability. The presence of Cd also triggers an increase in H_2_O_2_ production within plant tissues. These findings underscore the oxidative stress imposed on plants by Cd, resulting in compromised cellular integrity and potential implications for plant health and growth [58].

In the present study, oxidative stress levels, measured by MDA and H_2_O_2_ concentrations, decreased in wheat plants treated with 0.5 mM salicylic acid (SA). In plants exposed to cadmium (Cd), supporting with SA increased both root and shoot antioxidant capacity. This enhancement helped mitigate oxidative damage to the membranes, as evidenced by reduced levels of reactive oxygen species (ROS) and malondialdehyde (MDA). Salicylic acid (SA) plays a crucial role in signaling and mitigating the damage caused by heavy metals in plants [59]. The impairment of cellular defense mechanisms may underlie the toxic symptoms observed with high concentrations of Cd. Plants utilize a sophisticated antioxidative defense system, where both enzymatic and non-enzymatic antioxidants collaborate to neutralize reactive oxygen species (ROS) [60]. This organized defense mechanism helps plants to counteract oxidative stress effectively, thereby minimizing the detrimental effects of heavy metal exposure [21]. The coordinated action of enzymatic and non-enzymatic antioxidants is crucial for scavenging reactive oxygen species (ROS) and maintaining physiological redox balance in organisms. Heavy metals like cadmium (Cd) increase antioxidant activity in various plant species, a vital response to stress. This highlights the importance of antioxidants in defending plants against oxidative stress induced by Cd. Salicylic acid (SA) aids in this defense by signaling pathways that mitigate Cd's harmful effects on cellular defense mechanisms. Together, enzymatic and non-enzymatic antioxidants form a well-coordinated defense system in plants, essential for preserving cellular integrity and overall health during stress [61]. This evidence clearly demonstrates that SA-treated plants significantly induced health-promoting compounds and enhanced the antioxidant capacities of seedlings [17]. Previous studies have suggested that heavy metals inhibit root and shoot growth by interfering with processes such as moisture regulation, cell division, respiration, activity of hydrolytic enzymes, and nutrient transport from storage tissues to the growing embryonic axis [62]. Our present work builds on this understanding by demonstrating that treatment with salicylic acid (SA) effectively enhances antioxidant capacities and promotes the production of health-promoting compounds in seedlings. This suggests that SA may mitigate the adverse effects of heavy metals on growth processes by bolstering plant defenses and supporting essential metabolic functions against oxidative stress. Salicylic acid (SA) treatment enhances the antioxidant activity of spinach leaves by boosting their phenolic content and enzyme levels [63]. Additionally, the data demonstrate that applying salicylic acid (SA) to bean seedlings partially reduced the toxicity of cadmium (Cd) during germination and early growth stages, potentially indicating a positive effect on yield [50]. Soaking bean seeds in a combination of cadmium (Cd) and salicylic acid (SA) improved the germination rate, reduced Cd accumulation, and mitigated oxidative damage at the membrane level. This suggests SA's potential beneficial effects during germination, especially in limiting cumulative damage from Cd, which is highly phytotoxic due to its interference with cell metabolism and regulation. These findings hint at SA's role not only in stress alleviation but also in potentially enhancing yield under Cd stress conditions [64]. In present work SA has been shown to increase wheat yield. Cd exposure negatively impacts crop productivity by disrupting essential physiological functions. However, SA promotes enhanced crop growth and higher yield in Cd-contaminated environments by improving nutrient absorption and activating stress-responsive genes. Additionally, SA-treated plants exhibit an increased number of grains per spike. According to [65] wheat plants under Cd stress, when treated with SA, produce the highest number of grains per spike.

**Figure 9:**
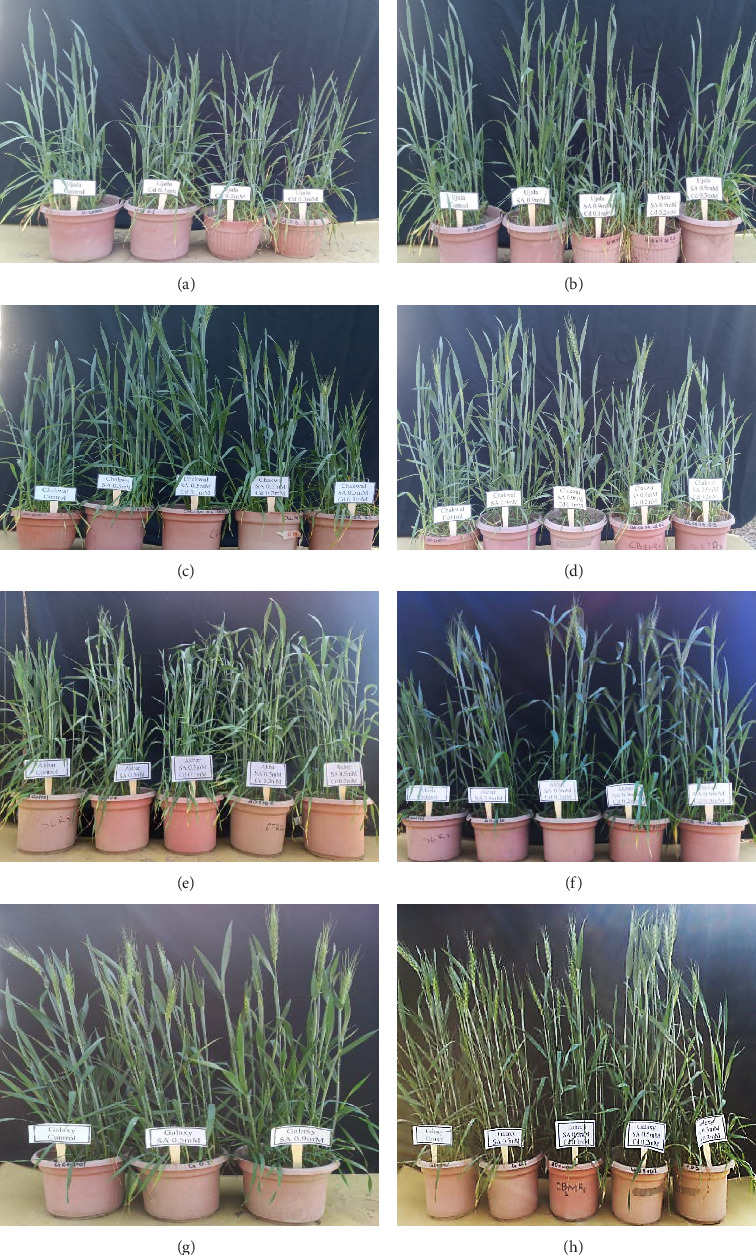
Showing different treatments of Cd in comparison with control in Ujala-16 (a), application of SA under Cd (b), 0.5 mM of SA under Cd stress in comparison with control of Chakwal-86 (c), 0.9 mM of SA under Cd stress in comparison with control of Chakwal-86 (d). 0.5 mM SA in comparison with control in Akbar-2019 (e), SA under Cd stress in comparison with control in Akbar-2019 (f), different treatments of SA in comparison with control in Galaxy-2013 (g), SA under Cd stress in comparison with control in Akbar-2019 (h).

Based on the findings of the present study, the application of salicylic acid (SA) significantly enhances the development and growth of wheat cultivars Akbar-2019, Galaxy-2013, Ujala-16, and Chakwal-86, particularly under cadmium (Cd) stress conditions. Akbar-2019 emerges as a particularly resilient cultivar, demonstrating superior morphological and biochemical characteristics compared to the others. Interestingly, the response to different SA concentrations (0.5 mM and 0.9 mM) varied between cultivars, with 0.5 mM SA alone and 0.9 mM SA under Cd stress proving most effective in promoting growth (Figure 9). The varying effects of SA concentration underscore the intricate interplay between SA signaling and Cd stress responses, necessitating further investigation. These findings offer valuable insights into the potential use of SA as a mitigating agent for Cd-induced stress in wheat cultivation, potentially revolutionizing crop management strategies in challenging environmental conditions. Continued research is essential to optimize SA application protocols and unravel the molecular mechanisms underlying its ability to enhance wheat productivity and resilience to heavy metal stress.

## 5. Conclusion

Salicylic acid (SA) significantly enhances wheat plants' defense mechanisms, particularly under cadmium (Cd) stress, which poses considerable risks to plant growth and human health. Our study reveals that while 0.5 mM SA is more effective than 0.9 mM SA under normal conditions, combining 0.9 mM SA with varying Cd concentrations yields the best growth and productivity under Cd stress. Exposure to 0.3 mM Cd negatively impacts all wheat varieties, but Akbar-2019 consistently exhibits superior growth and yield under Cd stress. In conclusion, SA proves to be a valuable tool for improving wheat resilience to Cd stress, supporting sustainable agriculture and environmental management. Insights gained from this study into the interactions between phytohormones, heavy metals, and crop responses are essential for developing practices to mitigate heavy metal contamination and enhance crop resilience.

## Data Availability

The data that support the findings of this study are available from the corresponding author upon reasonable request.

## Supporting Information

Supporting table 1: Impact of Cd and SA on morphological parameters of wheat. Supporting table 2: Impact of Cd and SA on relative water contents, total chlorophyl contents, carotenoid contents, no. of grains, grains fresh weight and grains dry weight of wheat. Supporting table 3: Impact of Cd and SA on TSP, CAT, APX, MDA and H2O2 of wheat.
