# Supplementary material for: Salicylic Acid Mitigates Cadmium Stress in Wheat: Experimental Insights Into Growth and Biochemical Parameters
Source: Scientifica (Cairo). 2024 Nov 30;2024:6887694. doi: 10.1155/sci5/6887694 (PMC11625088; doi:10.1155/sci5/6887694)
Supplement: Supporting Information — Additional supporting information can be found online in the Supporting Information section. [file 6887694.f1.docx]

**Supplementary table 1: Impact of Cd and SA on morphological parameters of wheat**

| **Cultivars** | **Treatments** | **Plant height (cm)** | **Total plant length (cm)** | **Leaf length (cm** | **Leaf width (cm** | **Leaf area (cm²)** | **Shoot FW (g)** | **Root FW (g)** | **Shoot DW (g)** | **Root DW (g)** |
| --- | --- | --- | --- | --- | --- | --- | --- | --- | --- | --- |
| **Chakwal-86** | **C0** | 25.367±0.463 | 32.8±0.907 | 10.767±0.088 | 0.83±0.033 | 8.973±0.379 | 3.077±0.014 | 1.437±0.008 | 1.077±o.014 | 0.437±0.008 |
|  | **Cd1** | 22.967±0.669 | 31.2±0.519 | 10.2±0.55 | 0.73±0.033 | 7.45±0.222 | 2.993±0.008 | 1.413±0.008 | 0.993±0.008 | 0.413±0.008 |
|  | **Cd2** | 22.8±0.519 | 29.03±0.272 | 9.467±0.318 | 0.63±0.033 | 5.987±0.285 | 2.71±0.049 | 1.337±0.008 | 0.71±0.049 | 0.337±0.008 |
|  | **Cd3** | 21.567±0.8006 | 27.2±0.4 | 8.63±0.384 | 0.53±0.0881 | 4.67±0.987 | 2.593±0.059 | 1.31±0.005 | 0.593±0.059 | 0.31±0.005 |
|  | **SA1** | 40.03±0.272 | 46.53±0.29 | 14.9±0.577 | 1.23±0.033 | 18.377±0.852 | 4.02±0.03 | 1.737±0.003 | 2.02±0.03 | 0.737±0.003 |
|  | **SA2** | 38.6±0.461 | 44.6±0.757 | 14.43±1.828 | 1.167±0.0881 | 17.16±3.33 | 4.0167±0.02 | 1.693±0.003 | 2.017±0.02 | 0.693±0.003 |
|  | **SA1Cd1** | 29.267±0.611 | 38.567±0.328 | 11.767±0.769 | 1.03±0.033 | 12.197±1.12 | 3.807±0.017 | 1.607±0.008 | 1.807±0.017 | 0.607±0.008 |
|  | **SA1Cd2** | 28±0.208 | 36.9±0.971 | 11.43±0.44 | 1±0.001 | 11.43±0.44 | 3.753±0.033 | 1.597±0.008 | 1.753±0.033 | 0.597±0.008 |
|  | **SA1Cd3** | 26.467±0.208 | 34±0.754 | 11.1±0.655 | 0.9±0.0577 | 9.93±0.417 | 3.683±0.008 | 1.557±0.041 | 1.683±0.008 | 0.557±0.041 |
|  | **SA2Cd1** | 34.6±0.692 | 43.3±0.290 | 14.3±1.747 | 1.13±0.0667 | 16.213±2.19 | 3.977±0.018 | 1.663±0.003 | 1.977±0.018 | 0.663±0.003 |
|  | **SA2Cd2** | 32.67±0.779 | 41.13±0.796 | 13.1±0.115 | 1.1±0.0577 | 14.417±0.833 | 3.923±0.029 | 1.623±0.003 | 1.923±0.029 | 0.623±0.003 |
|  | **SA2Cd3** | 31.167±0.384 | 39.5±0.889 | 12±0.404 | 1.067±0.0881 | 12.78±0.995 | 3.857±0.008 | 1.613±0.003 | 1.857±0.008 | 0.613±0.003 |
| **Ujala-16** | **C0** | 30.67±0.674 | 36.63±0.088 | 12.63±0.648 | 1±0.0577 | 12.683±1.22 | 3.597±0.008 | 1.47±0.015 | 1.597±0.008 | 0.47±0.015 |
|  | **Cd1** | 28.53±0.409 | 35.3±0.417 | 10.767±0.825 | 0.967±0.12 | 10.21±0.43 | 3.537±0.003 | 1.443±0.003 | 1.537±0.003 | 0.443±0.003 |
|  | **Cd2** | 26.867±0.617 | 33.367±0.284 | 9.67±0.233 | 0.93±0.0881 | 9.003±0.782 | 3.377±0.003 | 1.437±0.055 | 1.377±0.003 | 0.437±0.055 |
|  | **Cd3** | 24.2±0.493 | 35.167±0.581 | 9.267±0.284 | 0.9±0.0577 | 8.343±0.631 | 3.077±0.003 | 1.43±0.011 | 1.077±0.003 | 0.43±0.011 |
|  | **SA1** | 44.467±0.296 | 49.63±0.328 | 19.1±0.55 | 1.367±0.033 | 26.1±0.956 | 4.103±0.027 | 1.747±0.003 | 2.103±0.027 | 0.747±0.003 |
|  | **SA2** | 42.13±1.18 | 48.467±0.731 | 19.03±0.352 | 1.3±0.033 | 25.373±0.705 | 4.023±0.0545 | 1.73±0.003 | 2.023±0.054 | 0.73±0.003 |
|  | **SA1Cd1** | 35.23±0.995 | 44.43±0.338 | 16.267±0.49 | 1.13±0.033 | 18.463±1.067 | 3.937±0.0218 | 1.647±0.003 | 1.937±0.021 | 0.647±0.003 |
|  | **SA1Cd2** | 33.33±0.656 | 42.2±0.251 | 14.8±0.608 | 1.067±0.0881 | 15.83±1.719 | 3.91±0.0692 | 1.627±0.006 | 1.91±0.069 | 0.627±0.006 |
|  | **SA1Cd3** | 31.767±0.554 | 41.03±0.731 | 13.53±0.433 | 1.03±0.033 | 13.96±0.238 | 3.753±0.0717 | 1.59±0.04 | 1.753±0.071 | 0.59±0.04 |
|  | **SA2Cd1** | 40.43±0.578 | 46.4±0.692 | 18.567±0.392 | 1.3±0.0577 | 24.093±0.602 | 4.01±0.005 | 1.707±0.008 | 2.01±0.005 | 0.707±0.008 |
|  | **SA2Cd2** | 38.2±1.21 | 45.6±0.964 | 18.3±0.608 | 1.23±0.033 | 22.567±0.909 | 3.98±0.04 | 1.653±0.003 | 1.98±0.04 | 0.653±0.003 |
|  | **SA2Cd3** | 36.33±0.405 | 45.167±0.635 | 17.23±0.635 | 1.2±0.0577 | 20.703±1.41 | 3.947±0.068 | 1.65±0.03 | 1.947±0.068 | 0.65±0.03 |
| **Galaxy** | **C0** | 38.167±0.726 | 45.167±0.726 | 14.7±0.781 | 1.1±0.0577 | 16.22±1.55 | 3.683±.033 | 1.763±0.029 | 1.683±0.033 | 0.763±0.029 |
|  | **Cd1** | 35.167±0.927 | 40.167±0.927 | 13.367±0.491 | 1.067±0.033 | 14.23±0.286 | 3.657±0.014 | 1.743±0.008 | 1.657±0.014 | 0.743±0.008 |
|  | **Cd2** | 34.767±0.788 | 39.767±0.788 | 12.83±0.284 | 1.03±0.033 | 13.28±0.73 | 3.557±0.02 | 1.657±0.101 | 1.557±0.02 | 0.657±0.101 |
|  | **Cd3** | 33.3±0.378 | 38.3±0.378 | 11.967±0.437 | 1±0.0577 | 11.96±0.786 | 3.523±0.0318 | 1.617±0.021 | 1.523±0.031 | 0.617±0.021 |
|  | **SA1** | 48.67±0.676 | 53.67±0.676 | 20.03±0.981 | 1.4±0.0577 | 28.15±2.515 | 4.12±0.0585 | 2.07±0.025 | 2.12±0.058 | 1.07±0.025 |
|  | **SA2** | 47.53±0.53 | 52.53±0.533 | 19.967±0.272 | 1.367±0.033 | 27.3±0.952 | 4.113±0.049 | 2.05±0.02 | 2.113±0.049 | 1.05±0.02 |
|  | **SA1Cd1** | 40.967±0.46 | 45.967±0.467 | 17.23±0.554 | 1.2±0.0577 | 20.6567±0.991 | 3.993±0.008 | 1.977±0.024 | 1.993±0.008 | 0.967±0.024 |
|  | **SA1Cd2** | 39.67±0.33 | 42.67±0.333 | 16.267±0.52 | 1.167±0.033 | 18.98±0.851 | 3.963±0.021 | 1.94±0.011 | 1.963±0.021 | 0.94±0.011 |
|  | **SA1Cd3** | 39.367±0.317 | 41.367±0.317 | 15.567±0.328 | 1.13±0.033 | 17.627±0.354 | 3.91±0.026 | 1.923±0.026 | 1.91±0.026 | 0.923±0.026 |
|  | **SA2Cd1** | 46.67±0.881 | 51.67±0.881 | 19.13±0.26 | 1.3±0.033 | 25.51±0.701 | 4.097±0.141 | 2.04±0.026 | 2.097±0.141 | 1.04±0.026 |
|  | **SA2Cd2** | 45.43±0.585 | 50.43±0.586 | 18.467±0.484 | 1.3±0.577 | 24.01±1.295 | 4.077±0.008 | 2.03±0.037 | 2.077±0.008 | 1.03±0.037 |
|  | **SA2Cd3** | 43.82±0.635 | 47.82±0.635 | 17.8±0.635 | 1.23±0.033 | 21.99±1.34 | 4.037±0.039 | 2.003±0.029 | 2.037±0.039 | 1.003±0.029 |
| **Akbar 2019** | **C0** | 42.967±0.753 | 48.967±0.753 | 14.767±0.284 | 1.167±0.033 | 17.2467±0.814 | 3.82±0.05 | 1.823±0.069 | 1.82±0.05 | 0.823±0.069 |
|  | **Cd1** | 38.67±0.783 | 44.67±0.783 | 14.467±0.384 | 1.13±0.033 | 16.3767±0.323 | 3.823±0.069 | 1.81±0.055 | 1.823±0.069 | 0.81±0.055 |
|  | **Cd2** | 35.4±0.404 | 41.4±0.404 | 13.23±1.126 | 1.1±0.001 | 14.5567±1.238 | 3.657±0.052 | 1.723±0.02 | 1.657±0.052 | 0.723±0.02 |
|  | **Cd3** | 34.267±0.554 | 39.267±0.554 | 12.93±0.536 | 1.067±0.033 | 13.81±0.848 | 3.59±0.035 | 1.69±0.03 | 1.59±0.035 | 0.69±0.03 |
|  | **SA1** | 60.5±0.405 | 71.167±0.405 | 22.3±0.536 | 1.467±0.033 | 32.73±0.595 | 4.173±0.04 | 2.113±0.024 | 2.173±0.04 | 1.113±0.024 |
|  | **SA2** | 58.13±0.366 | 65.13±0.366 | 21.3±0.272 | 1.43±0.033 | 30.59±1.02 | 4.147±0.024 | 2.103±0.027 | 2.147±0.024 | 1.103±0.027 |
|  | **SA1Cd1** | 50.03±0.783 | 59.93±3.371 | 17.57±2.493 | 1.3±0.0577 | 22.957±3.65 | 4.03±0.056 | 2.03±0.056 | 2.03±0.056 | 1.03±0.057 |
|  | **SA1Cd2** | 50.4±0.665 | 57.4±0.665 | 16.6±0.68 | 1.267±0.033 | 20.993±0.603 | 4.007±0.043 | 2.007±0.043 | 2.007±0.043 | 1.007±0.043 |
|  | **SA1Cd3** | 44.567±0.753 | 51.567±0.29 | 15.9±1.053 | 1.23±0.067 | 19.53±1.181 | 3.937±0.008 | 1.927±0.021 | 1.937±0.008 | 0.927±0.021 |
|  | **SA2Cd1** | 56.5±0.665 | 63.5±1.266 | 20.53±0.68 | 1.4±0.0577 | 28.803±1.955 | 4.13±0.175 | 2.1±0.057 | 2.13±0.175 | 1.1±0.057 |
|  | **SA2Cd2** | 51.43±0.12 | 58.43±0.12 | 19.13±0.23 | 1.367±0.067 | 26.12±0.971 | 4.1±0.07 | 2.067±0.058 | 2.1±0.07 | 1.067±0.058 |
|  | **SA2Cd3** | 51.13±0.409 | 58.13±0.409 | 18.03±0.61 | 1.3±0.033 | 24.05±1.064 | 4.103±0.029 | 2.037±0.039 | 2.103±0.029 | 1.037±0.039 |

**Supplementary table 2: Impact of Cd and SA on relative water contents, total chlorophyl contents, carotenoid contents, no. of grains, grains fresh weight and grains dry weight of wheat**

| **Cultivars** | **Treatments** | **RWC (%)** | **Total Chlorophyll content (µg g^-1^ FW)** | **Car content (µg g^-1^FW)** | **No. of Grains** | **Grains FW (g)** | **Grains DW (g)** |
| --- | --- | --- | --- | --- | --- | --- | --- |
| **Chakwal-86** | **C0** | 86.48 ± 0.022 | 0.034 ± 0.0015 | 158.3 ± 0.333 | 20.67 ± 0.67 | 0.537 ± 0.0067 | 0.517 ± 0.0067 |
|  | **Cd1** | 86.27 ± 0.36 | 0.032 ± 0.00034 | 157.67 ± 0.88 | 19 ± 0.58 | 0.41 ± 0.01 | 0.39 ± 0.01 |
|  | **Cd2** | 85.72 ± 0.027 | 0.0307 ± 0.00088 | 156.3 ± 0.333 | 17.67 ± 0.33 | 0.38 ± 0.01 | 0.36 ± 0.01 |
|  | **Cd3** | 85.6 ± 0.022 | 0.029 ± 0.000882 | 154.67 ± 0.333 | 16 ± 0.58 | 0.357 ± 0.009 | 0.337 ± 0.009 |
|  | **SA1** | 94.55 ± 0.04 | 0.051 ± 0.000577 | 171.3 ± 0.88 | 31.33 ± 0.33 | 1.25 ± 0.0033 | 1.234 ± 0.0058 |
|  | **SA2** | 92.06 ± 0.02 | 0.050 ± 0.000882 | 170.3 ± 0.333 | 30.33 ± 0.33 | 1.236 ± 0.0067 | 1.2 ± 0.0034 |
|  | **SA1Cd1** | 87.58 ± 0.057 | 0.0427 ± 0.000882 | 163.3 ± 0.88 | 23.33 ± 0.33 | 0.853 ± 0.009 | 0.83 ± 0.0058 |
|  | **SA1Cd2** | 87.1 ± 0.037 | 0.039 ± 0.00115 | 161.3 ± 0.333 | 23 ± 0.58 | 0.85 ± 0.01 | 0.827 ± 0.01 |
|  | **SA1Cd3** | 86.82 ± 0.015 | 0.036 ± 0.00208 | 159.3 ± 0.88 | 21.67 ± 0.67 | 0.83 ± 0.01 | 0.81 ± 0.01 |
|  | **SA2Cd1** | 89.43 ± 0.044 | 0.049 ± 0.000882 | 168.67 ± 0.67 | 27 ± 0.58 | 1.15 ± 0.01 | 1.107 ± 0.009 |
|  | **SA2Cd2** | 89.37 ± 0.022 | 0.047 ± 0.0012 | 167 ± 0.58 | 25 ± 0.58 | 0.88 ± 0.01 | 0.857 ± 0.01 |
|  | **SA2Cd3** | 88.28 ± 0.049 | 0.045 ± 0.00145 | 164.67 ± 0.67 | 24 ± 0.58 | 0.873 ± 0.009 | 0.85 ± 0.01 |
| **Ujala-16** | **C0** | 86.51 ± 0.037 | 0.044 ± 0.00034 | 158.67 ± 0.33 | 24.63 ± 0.33 | 0.873 ± 0.0067 | 0.85 ± 0.0067 |
|  | **Cd1** | 86.30 ± 0.033 | 0.0417 ± 0.00233 | 158 ± 0.58 | 23.067 ± 0.33 | 0.86 ± 0.0058 | 0.84 ± 0.0058 |
|  | **Cd2** | 85.94 ± 0.03 | 0.0387 ± 0.00273 | 156.67 ± 0.33 | 22 ± 0.58 | 0.837 ± 0.009 | 0.817 ± 0.009 |
|  | **Cd3** | 85.6 ± 0.035 | 0.034 ± 0.00176 | 155 ± 0.58 | 19.33 ± 0.33 | 0.757 ± 0.0067 | 0.737 ± 0.0067 |
|  | **SA1** | 94.91 ± 0.317 | 0.051 ± 0.00034 | 172 ± 0.58 | 45 ± 0.58 | 1.323 ± 0.009 | 1.3 ± 0.0058 |
|  | **SA2** | 93.01 ± 0.038 | 0.0507 ± 0.000882 | 171 ± 0.58 | 41 ± 0.58 | 1.283 ± 0.01 | 1.253 ± 0.01 |
|  | **SA1Cd1** | 87.91 ± 0.33 | 0.0477 ± 0.000882 | 163.67 ± 0.67 | 29.33 ± 0.33 | 1.08 ± 0.0067 | 1.053 ± 0.009 |
|  | **SA1Cd2** | 87.69 ± 0.59 | 0.047 ± 0.00058 | 161.67 ± 0.33 | 27 ± 0.58 | 1.03 ± 0.01 | 1.01 ± 0.01 |
|  | **SA1Cd3** | 86.95 ± 0.046 | 0.0457 ± 0.000882 | 160 ± 0.58 | 24.67 ± 0.88 | 0.99 ± 0.01 | 0.97 ± 0.01 |
|  | **SA2Cd1** | 89.56 ± 0.11 | 0.05 ± 0.00115 | 169 ± 0.58 | 37 ± 0.58 | 1.203 ± 0.009 | 1.183 ± 0.009 |
|  | **SA2Cd2** | 89.46 ± 0.033 | 0.049 ± 0.000882 | 167.3 ± 0.33 | 35 ± 0.58 | 1.17 ± 0.01 | 1.143 ± 0.01 |
|  | **SA2Cd3** | 89.37 ± 0.079 | 0.0487 ± 0.000882 | 165 ± 0.58 | 32 ± 0.58 | 1.11 ± 0.0058 | 1.09 ± 0.0058 |
| **Galaxy** | **C0** | 87.32 ± 0.64 | 0.0477 ± 0.0012 | 161.3 ± 0.33 | 36.33 ± 0.33 | 1.563 ± 0.0067 | 1.534 ± 0.0067 |
|  | **Cd1** | 87.16 ± 0.018 | 0.0473 ± 0.000882 | 158.67 ± 2.33 | 35.67 ± 0.33 | 1.56 ± 0.0058 | 1.53 ± 0.0058 |
|  | **Cd2** | 86.18 ± 0.25 | 0.047 ± 0.00058 | 157.3 ± 2.02 | 34 ± 0.58 | 1.537 ± 0.009 | 1.507 ± 0.009 |
|  | **Cd3** | 86.08 ± 0.35 | 0.047 ± 0.000882 | 156.67 ± 0.88 | 31.33 ± 0.33 | 1.457 ± 0.0067 | 1.427 ± 0.0067 |
|  | **SA1** | 96.58 ± 0.033 | 0.0527 ± 0.00034 | 172.67 ± 0.88 | 57 ± 0.58 | 2.023 ± 0.009 | 1.993 ± 0.009 |
|  | **SA2** | 94.58 ± 0.58 | 0.0517 ± 0.00034 | 171.67 ± 0.67 | 53 ± 0.58 | 1.973 ± 0.01 | 1.943 ± 0.01 |
|  | **SA1Cd1** | 89.07 ± 0.013 | 0.0487 ± 0.000882 | 166.3 ± 0.88 | 41.33 ± 0.33 | 1.78 ± 0.0067 | 1.747 ± 0.0067 |
|  | **SA1Cd2** | 88.12 ± 0.77 | 0.048 ± 0.00088**2** | 165 ± 2.08 | 39 ± 0.58 | 1.73 ± 0.01 | 1.7 ± 0.01 |
|  | **SA1Cd3** | 87.86 ± 0.029 | 0.048 ± 0.00115 | 163.3 ± 0.67 | 36.67 ± 0.88 | 1.69 ± 0.01 | 1.66 ± 0.01 |
|  | **SA2Cd1** | 89.85 ± 0.037 | 0.050 ± 0.0013 | 171 ± 1.15 | 49 ± 0.58 | 1.9 ± 0.009 | 1.873 ± 0.009 |
|  | **SA2Cd2** | 89.60 ± 0.087 | 0.0497 ± 0.0012 | 169.67 ± 1.76 | 47 ± 0.58 | 1.87 ± 0.01 | 1.84 ± 0.01 |
|  | **SA2Cd3** | 89.52 ± 0.102 | 0.049 ± 0.000882 | 168 ± 0.578 | 44 ± 0.58 | 1.81 ± 0.0058 | 1.78 ± 0.0058 |
| **Akbar 2019** | **C0** | 87.51 ± 0.037 | 0.048 ± 0.00058 | 162.67 ± 0.88 | 40.33 ± 0.33 | 1.963 ± 0.0067 | 1.934 ± 0.0067 |
|  | **Cd1** | 87.38 ± 0.19 | 0.0477 ± 0.0012 | 161.67 ± 1.86 | 39.67 ± 0.33 | 1.96 ± 0.0058 | 1.93 ± 0.0058 |
|  | **Cd2** | 87.18 ± 0.25 | 0.0473 ± 0.000882 | 160.3 ± 1.45 | 38 ± 0.58 | 1.937 ± 0.009 | 1.907 ± 0.009 |
|  | **Cd3** | 87.08 ± 0.307 | 0.047 ± 0.00058 | 157.67 ± 1.45 | 35.33 ± 0.33 | 1.857 ± 0.0067 | 1.827 ± 0.0067 |
|  | **SA1** | 98.41 ± 0.00088 | 0.0533 ± 0.000882 | 173.3 ± 1.45 | 61 ± 0.58 | 2.423 ± 0.009 | 2.393 ± 0.009 |
|  | **SA2** | 96.58 ± 0.578 | 0.052 ± 0.00058 | 172.67 ± 0.67 | 57 ± 0.58 | 2.373 ± 0.01 | 2.343 ± 0.01 |
|  | **SA1Cd1** | 89.66 ± 0.33 | 0.049 ± 0.00115 | 167.3 ± 0.88 | 45.33 ± 0.33 | 2.177 ± 0.0067 | 2.147 ± 0.0067 |
|  | **SA1Cd2** | 88.99 ± 0.016 | 0.0487 ± 0.000882 | 165.3 ± 1.2 | 43 ± 0.58 | 2.13 ± 0.01 | 2.1 ± 0.01 |
|  | **SA1Cd3** | 88.65 ± 0.658 | 0.0483 ± 0.00145 | 164.3 ± 0.88 | 40.67 ± 0.88 | 2.09 ± 0.01 | 2.06 ± 0.01 |
|  | **SA2Cd1** | 90.37 ± 0.048 | 0.0513 ± 0.00034 | 171.66 ± 1.15 | 53 ± 0.58 | 2.3 ± 0.009 | 2.273 ± 0.009 |
|  | **SA2Cd2** | 90.17 ± 0.037 | 0.0507 ± 0.00088**2** | 170.3 ± 0.67 | 51 ± 0.58 | 2.27 ± 0.01 | 2.24 ± 0.01 |
|  | **SA2Cd3** | 89.99 ± 0.033 | 0.0497 ± 0.00034 | 169 ± 1.15 | 48 ± 0.58 | 2.21 ± 0.0058 | 2.18 ± 0.0058 |

**Supplementary table 3: Impact of Cd and SA on TSP, CAT, APX, MDA and H_2_O_2_ of wheat**

| **Cultivars** | **Treatments** | **TSP (mg g^-1^ FW)** | **CAT (U g^-1^ P)** | **APX (U g^-1^ P)** | **MDA (nmol g^-1^ FW)** | **H_2_O_2_ (nmol g^-1^ FW)** |
| --- | --- | --- | --- | --- | --- | --- |
| **Chakwal-86** | **C0** | 0.73 ± 0.012 | 0.123 ± 0.0034 | 0.047 ± 0.0067 | 53.43 ± 0.273 | 14.5 ± 0.305 |
|  | **Cd1** | 0.727 ± 0.0145 | 0.12 ± 0.0058 | 0.043 ± 0.009 | 53.767 ± 0.09 | 15.167 ± 0.27 |
|  | **Cd2** | 0.65 ± 0.0058 | 0.117 ± 0.0433 | 0.037 ± 0.0034 | 54.1 ± 0.252 | 15.13 ± 0.28 |
|  | **Cd3** | 0.55 ± 0.0057 | 0.11 ± 0.0058 | 0.02 ± 0.0058 | 55 ± 0.265 | 15.467 ± 0.62 |
|  | **SA1** | 0.89 ± 0.0115 | 0.17 ± 0.0058 | 0.08 ± 0.0058 | 48.7 ± 0.635 | 11.5 ± 0.83 |
|  | **SA2** | 0.887 ± 0.012 | 0.167 ± 0.0089 | 0.073 ± 0.009 | 48.03 ± 0.338 | 11.73 ± 0.09 |
|  | **SA1Cd1** | 0.83 ± 0.024 | 0.147 ± 0.0089 | 0.0567 ± 0.0034 | 52.1 ± 0.252 | 13.5 ± 0.305 |
|  | **SA1Cd2** | 0.8 ± 0.026 | 0.143 ± 0.0034 | 0.053 ± 0.0034 | 52.767 ± 0.524 | 13.83 ± 0.067 |
|  | **SA1Cd3** | 0.767 ± 0.021 | 0.13 ± 0.0034 | 0.05 ± 0.0058 | 53.767 ± 0.61 | 14.167 ± 0.27 |
|  | **SA2Cd1** | 0.883 ± 0.009 | 0.163 ± 0.0089 | 0.07 ± 0.0058 | 50.03 ± 0.338 | 12.067 ± 0.273 |
|  | **SA2Cd2** | 0.853 ± 0.019 | 0.157 ± 0.0034 | 0.067 ± 0.009 | 50.367 ± 0.285 | 12.4 ± 0.36 |
|  | **SA2Cd3** | 0.8367 ± 0.012 | 0.15 ± 0.0058 | 0.06 ± 0.0058 | 51.03 ± 0.384 | 13.167 ± 0.27 |
| **Ujala-16** | **C0** | 0.78 ± 0.0088 | 0.13 ± 0.0058 | 0.053 ± 0.0034 | 52.43 ± 0.273 | 13.5 ± 0.305 |
|  | **Cd1** | 0.747 ± 0.0273 | 0.127 ± 0.0034 | 0.047 ± 0.009 | 52.767 ± 0.09 | 14.167 ± 0.37 |
|  | **Cd2** | 0.69 ± 0.0088 | 0.123 ± 0.0034 | 0.04 ± 0.0058 | 53.1 ± 0.252 | 14.467 ± 0.38 |
|  | **Cd3** | 0.58 ± 0.0219 | 0.113 ± 0.0034 | 0.0267 ± 0.0034 | 54 ± 0.265 | 14.8 ± 0.058 |
|  | **SA1** | 0.9 ± 0.0153 | 0.18 ± 0.0034 | 0.083 ± 0.0034 | 47.7 ± 0.635 | 10.5 ± 0.305 |
|  | **SA2** | 0.89 ± 0.0115 | 0.173 ± 0.0034 | 0.08 ± 0.0058 | 48.367 ± 0.338 | 10.73 ± 0.09 |
|  | **SA1Cd1** | 0.84 ± 0.0176 | 0.15 ± 0.0115 | 0.063 ± 0.0067 | 51.1 ± 0.252 | 12.5 ± 0.305 |
|  | **SA1Cd2** | 0.82 ± 0.0088 | 0.147 ± 0.0034 | 0.06 ± 0.0058 | 51.767 ± 0.524 | 12.83 ± 0.067 |
|  | **SA1Cd3** | 0.81 ± 0.0058 | 0.137 ± 0.0034 | 0.0567 ± 0.0034 | 52.1 ± 0.252 | 13.167 ± 0.27 |
|  | **SA2Cd1** | 0.887 ± 0.012 | 0.17 ± 0.0034 | 0.073 ± 0.0034 | 49.03 ± 0.338 | 11.067 ± 0.273 |
|  | **SA2Cd2** | 0.86 ± 0.015 | 0.16 ± 0.0058 | 0.07 ± 0.01 | 49.367 ± 0.285 | 11.4 ± 0.36 |
|  | **SA2Cd3** | 0.847 ± 0.021 | 0.153 ± 0.009 | 0.067 ± 0.009 | 50.03 ± 0.384 | 12.167 ± 0.27 |
| **Galaxy** | **C0** | 0.787 ± 0.0088 | 0.137 ± 0.0034 | 0.0567 ± 0.0034 | 50.93 ± 0.273 | 12 ± 0.305 |
|  | **Cd1** | 0.75 ± 0.0058 | 0.13 ± 0.0034 | 0.053 ± 0.0034 | 51.267 ± 0.09 | 12.67 ± 0.37 |
|  | **Cd2** | 0.7 ± 0.0058 | 0.127 ± 0.0034 | 0.05 ± 0.0058 | 51.6 ± 0.252 | 12.967 ± 0.38 |
|  | **Cd3** | 0.59 ± 0.02 | 0.117 ± 0.0088 | 0.0367 ± 0.009 | 52.5 ± 0.265 | 13.3 ± 0.058 |
|  | **SA1** | 0.92 ± 0.01 | 0.18 ± 0.0058 | 0.0867 ± 0.0034 | 46.2 ± 0.635 | 9 ± 0.305 |
|  | **SA2** | 0.917 ± 0.012 | 0.173 ± 0.0034 | 0.083 ± 0.0034 | 46.867 ± 0.338 | 9.23 ± 0.09 |
|  | **SA1Cd1** | 0.847 ± 0.0328 | 0.1533 ± 0.0034 | 0.067 ± 0.0034 | 49.6 ± 0.252 | 11 ± 0.305 |
|  | **SA1Cd2** | 0.83 ± 0.0115 | 0.15 ± 0.0115 | 0.063 ± 0.0034 | 50.267 ± 0.524 | 11.3 ± 0.067 |
|  | **SA1Cd3** | 0.81 ± 0.009 | 0.14 ± 0.0058 | 0.06 ± 0.0058 | 50.6 ± 0.252 | 11.67 ± 0.27 |
|  | **SA2Cd1** | 0.907 ± 0.0034 | 0.17 ± 0.0058 | 0.0767 ± 0.0067 | 47.53 ± 0.338 | 9.57 ± 0.273 |
|  | **SA2Cd2** | 0.9 ± 0.01 | 0.163 ± 0.0034 | 0.073 ± 0.0034 | 47.867 ± 0.285 | 9.9 ± 0.36 |
|  | **SA2Cd3** | 0.88 ± 0.0176 | 0.157 ± 0.0034 | 0.07 ± 0.0058 | 48.53 ± 0.384 | 10.67 ± 0.27 |
| **Akbar 2019** | **C0** | 0.83 ± 0.012 | 0.153 ± 0.0034 | 0.0727 ± 0.0027 | 50.03 ± 0.273 | 11.1 ± 0.305 |
|  | **Cd1** | 0.783 ± 0.0338 | 0.14 ± 0.0058 | 0.0687 ± 0.00145 | 50.367 ± 0.09 | 11.767 ± 0.37 |
|  | **Cd2** | 0.75 ± 0.0058 | 0.13 ± 0.0058 | 0.068 ± 0.0009 | 50.7 ± 0.252 | 12.067 ± 0.38 |
|  | **Cd3** | 0.617 ± 0.028 | 0.123 ± 0.009 | 0.043 ± 0.0034 | 51.6 ± 0.265 | 12.4 ± 0.058 |
|  | **SA1** | 0.947 ± 0.0185 | 0.187 ± 0.0034 | 0.089 ± 0.00034 | 45.3 ± 0.635 | 8.1 ± 0.305 |
|  | **SA2** | 0.94 ± 0.02 | 0.18 ± 0.0058 | 0.0857 ± 0.0009 | 45.967 ± 0.338 | 8.3 ± 0.09 |
|  | **SA1Cd1** | 0.877 ± 0.04 | 0.16 ± 0.0058 | 0.08 ± 0.0015 | 48.7 ± 0.252 | 10.1 ± 0.305 |
|  | **SA1Cd2** | 0.87 ± 0.043 | 0.157 ± 0.00334 | 0.0793 ± 0.0009 | 49.367 ± 0.524 | 10.43 ± 0.067 |
|  | **SA1Cd3** | 0.83 ± 0.062 | 0.1533 ± 0.0034 | 0.076 ± 0.0025 | 49.7 ± 0.252 | 10.767 ± 0.27 |
|  | **SA2Cd1** | 0.937 ± 0.027 | 0.173 ± 0.0034 | 0.0837 ± 0.00034 | 46.63 ± 0.338 | 8.67 ± 0.273 |
|  | **SA2Cd2** | 0.92 ± 0.009 | 0.17 ± 0.0058 | 0.0823 ± 0.0009 | 46.967 ± 0.285 | 9 ± 0.36 |
|  | **SA2Cd3** | 0.9167 ± 0.018 | 0.163 ± 0.0034 | 0.0817 ± 0.00034 | 47.63 ± 0.384 | 9.767 ± 0.27 |
